# Influence of Obesity on the Organization of the Extracellular Matrix and Satellite Cell Functions After Combined Muscle and Thorax Trauma in C57BL/6J Mice

**DOI:** 10.3389/fphys.2020.00849

**Published:** 2020-07-28

**Authors:** Adrian Gihring, Fabian Gärtner, Congxing Liu, Markus Hoenicka, Martin Wabitsch, Uwe Knippschild, Pengfei Xu

**Affiliations:** ^1^Department of General and Visceral Surgery, Surgery Center, Ulm University Medical Center, Ulm, Germany; ^2^Department of Cardio-Thoracic and Vascular Surgery, Ulm University Medical Center, Ulm, Germany; ^3^Division of Pediatric Endocrinology and Diabetes, Department of Pediatrics and Adolescent Medicine, Ulm University Medical Center, Ulm, Germany

**Keywords:** diet-induced obesity, combined blunt trauma, regeneration, ECM organization, satellite cell physiology

## Abstract

Obesity has been described as a major factor of health risk in modern society. Next to intricately linked comorbidities like coronary artery disease or diabetes, an influence of obesity on regeneration after muscle injury has been described previously. However, the influence of obesity on tissue regeneration in a combined trauma, merging the more systemic influence of a blunt lung trauma and the local blunt muscle trauma, has not been investigated yet. Therefore, the aim of this study was to investigate the influence of obesity on regeneration in a mouse model that combined both muscle and thorax trauma. Using gene expression analysis, a focus was put on the structure as well as the organization of the extracellular matrix and on functional satellite cell physiology. An increased amount of debris in the lung of obese mice compared to normal weight mice up to 192 h after combined trauma based on visual assessment can be reported which is accompanied by a decreased response of *Mmp2* in obese mice. Additionally, a delayed and elongated response of inhibitor genes like *Timp1* has been revealed in obese mice. This elongated response to the trauma in obese mice can also be seen in plasma based on increased levels of pro-inflammatory chemo- and cytokines (IL-6, MCP-1, and IL 23) 192 h post trauma. In addition to changes in the lung, morphological analysis of the injured *extensor iliotibialis anticus* of the left hind leg in lean and diet-induced obese mice revealed deposition of fat in the regenerating muscle in obese animals hindering the structure of a compact muscle. Additionally, decreased activation of satellite cells and changes in organization and build-up of the ECM could be detected, finally leading to a decreased stability of the regenerated muscle in obese mice. Both factors contribute to an attenuated response to the trauma by obese mice which is reflected by a statistically significant decrease in muscle force of obese mice compared to lean mice 192 h post trauma induction.

## Graphical Abstract

The general workflow of the study is shown starting with the set-up of the trauma resulting with the collection of the tissue. The collected tissues from the injured or control muscle (1), lung tissue (2), and blood (3) were further investigated using various methods listed in this graphical abstract.

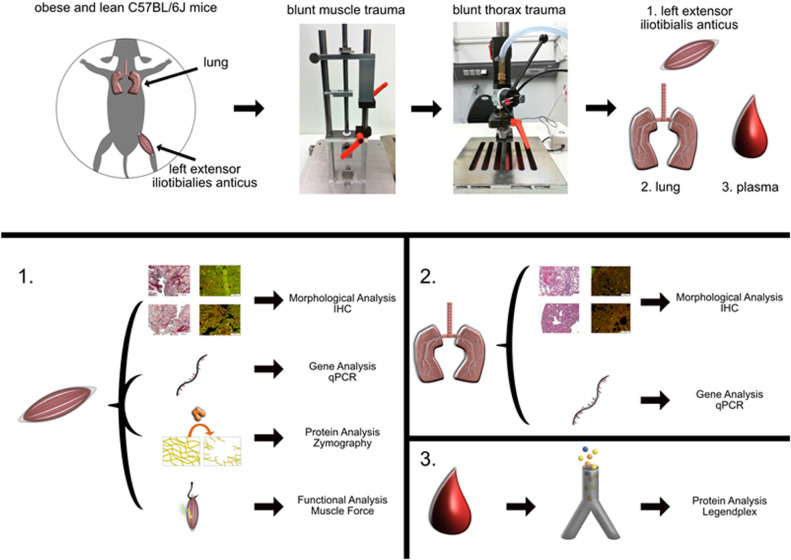

## Introduction

The cases of severe obesity are still rising, and a stagnation is not likely within the next decades (Smith and Smith, 2016). Apart from serious negative effects on social acceptance and psychology, obesity has severe effects on physical health and is associated with a massive economic burden (Tremmel et al., 2017). Understanding how obesity influences the development and outcome of common diseases on a molecular level is indispensable for an improved and adapted treatment. Adipose tissue, massively accumulated in obesity, is seen as an endocrine organ that releases various adipocytokines like leptin or adiponectin, free-fatty acids, and sex steroids. Since tissue resident macrophages release pro-inflammatory cytokines like tumor necrosis factor alpha (TNF-α), Interleukin 6 (IL-6), Interleukin 1β (IL-1β), or monocyte chemoattractant protein-1 (MCP-1) (Kershaw and Flier, 2004), obesity is seen as a state of chronic inflammation thereby not only influencing the immune response but also several metabolic processes like energy homeostasis, glucose and lipid metabolism (Coelho et al., 2013). Therefore, the issue of obesity has become a field of focus in the trauma research as well. It has been shown that the treatment and the life expectancy after polytraumatic injuries is highly influenced by additional risk factors like obesity (Hoffmann et al., 2012). However, the influence of obesity on the outcome of combined trauma injuries has not been sufficiently investigated (Rau et al., 2017).

It is known that obesity is associated with increased mortality and morbidity in trauma patients which can most likely be linked with a higher incidence of multiple organ failure, altered posttraumatic inflammatory response and pulmonary complications like pneumonia (Hoffmann et al., 2012; Andruszkow et al., 2013; Mica et al., 2013; Gray and Dieudonne, 2018; Spitler et al., 2018), finally resulting in a significantly increased hospitalization with intensive care unit treatment (Licht et al., 2015). Although it has been shown that there exists a negative correlation between obesity and the outcome after combined trauma injury, the molecular mechanisms remain unclear. Besides adverse effects and complication in the lung, obesity has also been shown to influence the regeneration of skeletal muscle (Brown and Kuk, 2015; D’Souza et al., 2015; Li et al., 2020; Verpoorten et al., 2020).

Obesity has a negative influence on muscle regeneration after blunt muscle trauma due to altered fatty acid metabolism and altered signaling pathways like sonic hedgehog signaling or insulin signaling (Akhmedov and Berdeaux, 2013; Fu et al., 2016; Werner et al., 2018; Xu P. et al., 2018). Satellite cells are key players in muscle regeneration after injury. Satellite cell population consists of paired-box protein Pax7 (PAX7) positive and myogenic factor 5 (MYF5) negative satellite stem cells and committed satellite cells (PAX7^+^, MYF5^+^) (Yin et al., 2013). Committed satellite cells are activated after injury, proliferate and finally differentiate into myotubes that mature to newly regenerated myofibers (Flamini et al., 2018). This process is characterized by the expression of the myogenic regulatory transcription factor myoblast determination protein 1 (MYOD) (Yamamoto et al., 2018) and myogenin (MYOG) (Meadows et al., 2011). Since it has been shown that obesity has an influence on the lung (pneumonia) and the skeletal muscle (altered fatty acid metabolism) in a multiple trauma situation, this study uses a combined trauma model to simulate a multiple traumatic injury by inducing a thorax trauma as well as a muscle trauma in the left hind leg. Herein, the lung trauma is supposed to lead to a more systemic response, since it has been shown that lung injury leads to a systemic release of pro-inflammatory cytokines (Hiraiwa and van Eeden, 2014).

Although tissue specific stem cells play a crucial role in the regeneration process of their domestic tissue, interconnected factors are additionally of great importance. The regulation and organization of ECM is a fundamental part of tissue regeneration (Kusindarta and Wihadmadyatami, 2018) and is necessary for the stability of the tissue during and after regeneration. ECM is the non-cellular component of the tissue and consists of different components including collagens, fibronectin, elastin, proteoglycans, and laminin (Frantz et al., 2010; Kular et al., 2014). Apart from a structural function the ECM is involved in the regulation of cell proliferation (Koohestani et al., 2013), cell adhesion (Schlie-Wolter et al., 2013), cell migration (Hartman et al., 2017), and cell differentiation (Smith et al., 2018; Novoseletskaya et al., 2019). Dysregulation and imbalance of the extracellular matrix is associated with cardiac dysfunction (Kim et al., 2000), destruction of cartilage in osteoarthritis (Shi et al., 2019), fibrosis formation (Herrera et al., 2018), and tumor development (Kessenbrock et al., 2010; Wei et al., 2017). Matrix metalloproteinases (MMPs) are enzymes that degrade components of the ECM and play a crucial role in the tissue regeneration process (Bellayr et al., 2009). They are involved in epithelial regeneration (Mohan et al., 2002), mediation and regulation of immune response (Fingleton, 2017; Xu S. et al., 2018) as well as in angiogenesis (Raza and Cornelius, 2000; Song et al., 2016; Sun et al., 2017) and ECM remodeling (Rohani and Parks, 2015). Altered ratios of MMPs and their inhibitors, referred to tissue inhibitors of matrix metalloproteinases (TIMPs), are often connected to impaired healing function (Mu et al., 2013).

Although it has been shown before that obesity has a negative effect on tissue regeneration (Brown and Kuk, 2015; D’Souza et al., 2015; Li et al., 2020; Verpoorten et al., 2020), the mechanisms and effects of diet induced obesity especially on ECM organization, regeneration of the tissue and on functional assessment of the regeneration has not been investigated yet. ECM, which contributes heavily on tissue structure and strength, is often overlooked in studies dealing with tissue regeneration. Therefore, this study addresses this issue focusing on ECM organization and regulation on gene, protein, and functional level after combined blunt lung and muscle trauma as it can be seen in the Graphical Abstract. The analysis of lung tissue after trauma revealed a prolonged and attenuated response to the trauma in obese mice after injury. This was detected in the gene expression analysis of *Timp1* and *Mmp2*, respectively. This shifted and elongated response to the trauma is also seen in the blood. Gene expression analysis of muscle tissue revealed changes in the ECM organization as well as the regeneration process. An increased gene expression of ECM structural proteins was detected in lean animals, which is accompanied by a bimodal response of *Lox* and *Timp1*. This hinds toward a hindered build-up of ECM in obese mice during the early and late response to the trauma. Additionally, a decreased activation of satellite cells as a response to traumatic injury (based on *Myog* and *Myod* expression) was observed, indicating a decreased regenerative capacity of obese animals. Both findings contribute to a decreased stability of the muscle in obese animals after trauma, which finally results in a decreased regeneration of the muscle force in obese mice 192 h after trauma induction. Furthermore, both findings, the prolonged response in obese animals as well as the decreased activation of satellite cells, are supported by the cyto- and chemokine profile in the blood.

## Materials and Methods

### Animal Model and Breeding

All mouse experiments were approved by the local and state authorities (Ulm University/license number: 1183) and carried out in accordance with local regulations and ARRIVE guidelines. In the context of the application for animal experiments, a power analysis was conducted, and the sample size was calculated (nominal power: 0.8, nominal alpha: 0.025). A mouse model consisting of male, non-genetically modified C57BL/6J mice linking diet-induced obesity (DIO) and a combined trauma injury was used for scientific investigation. Parental animals received either a normal diet (ND, 10% kcal fat; DI12450) or a high-fat diet (HFD, 60% kcal fat, DI12492) 1 week prior to breeding to make the litter higher susceptible for DIO and to influence prenatal development (Tamashiro et al., 2009). Litters were weaned after 3 weeks and received the parental diet. Rearing conditions include a 12 h light/dark cycle at 22.5 ± 1°C with unlimited access to food and water. To assess the success of DIO, body weight was determined. For induction of the multiple traumatic injury 16 ± 1 week old lean and obese male mice were randomly grouped into control and trauma.

### Induction of Combined Trauma

Control and trauma mice were treated equally before and after trauma induction with the exception that control mice did not receive a traumatic injury. Anesthesia was carried out in an anesthesia tube using a mixture of 2.5 vol% sevoflurane and 97.5 vol% oxygen. Buprenorphine (0.3 mg/mL) was injected subcutaneously with ongoing anesthesia using a rodent anesthesia mask. Left upper leg and chest area of mice were shaved. To simulate a combined-trauma mouse model a blunt thorax trauma was induced after a blunt skeletal muscle trauma. Muscle trauma was induced in the muscle of the left hind leg *extensor iliotibialis anticus* using a drop tower apparatus described previously (Werner et al., 2018; Xu P. et al., 2018). The leg was fixed between scaffold and wedge. A weight with a mass of 40 g was dropped from a height of 104 cm leading to a blunt muscle injury evoked by the penetrating wedge. The depth of penetration was limited by a spacer (3 mm) to prevent bone fractures. Subsequent, thorax trauma was induced using a blast wave generator described previously (Knoferl et al., 2004; Kalbitz et al., 2017). Sternum was centrally placed under the cylinder. A high-speed valve which can be triggered manually was connected to a gas cylinder containing compressed air with a pressure reducer adjusted to 13 bar. Tissue samples of lung and muscle were collected from control and trauma mice 1, 6, 24, 72, and 192 h post trauma.

### Morphological Evaluation and Collagen Quantification

Muscle tissue of the *extensor iliotibialis anticus* of the left leg and lung tissue from untreated or injured normal weight (*n* = 6) and obese mice (*n* = 6) were collected at different time points. Paraffin-embedded, 5 μm thick cross-sections of skeletal muscle and lung tissue were dried for 24 h at 40°C with subsequent deparaffinization in Roti-Histol^®^ and hydration in decreasing alcohol series before having been used for staining with Hematoxylin and eosin (HE) and Sirius red (SR).

#### HE Staining

Specimens were stained for 3 min in hematoxylin (Gill No. 3, Sigma-Aldrich, Munich, Germany), washed 2 times with H_2_O followed by differentiating in acidic alcohol (1% HCl in ethanol) for 3 s and bluing under running tap water for 10 min. Counterstaining was conducted with eosin (Eosin Y, Sigma-Aldrich, Munich, Germany) for 10 s. Dehydration was performed in increasing alcohol series and Roti-Histol^®^. Finally, sections were mounted with Entellan^®^. One area per animal was evaluated based on the region of trauma. Six animals were used for each group based on the power analysis conducted for the animal experiment application (license number: 1183). Pictures were taken with the UC30 color camera at X10 and X40 magnification. All stainings were acquired with an Olympus IX81 and analyzed with the Olympus software cellSens Dimensions 2.3 (Build 18987).

#### SR Staining

Deparaffinized sections were stained in SR solution (0.1% in aqueous saturated picric acid) for 1 h, differentiated in 0.5% acetic acid and counterstained with hematoxylin for 10 min with subsequent differentiation and bluing as previously described (Xu P. et al., 2018). Dehydration was performed in increasing alcohol series and Roti-Histol^®^. Sections were covered with Entellan^®^. One area per animal was evaluated based on the region of trauma. Six animals were used for each group based on the power analysis conducted for the animal experiment application (license number: 1183). Pictures were taken with the UC30 color camera at X10 and X40 magnification. All stainings were acquired with an Olympus IX81 and analyzed with the Olympus software cellSens Dimensions 2.3 (Build 18987).

#### Collagen Quantification

SR stained tissue section were analyzed using the fluorescence based method described by Vogel et al. (2015) based on the analysis of the collagen content within picrosirius red stained tissue with fluorescence microscopy. This was adapted and the collagen content was calculated and normalized to the total area of tissue, which was calculated by the addition of collagen and live cell content. One area per animal was evaluated based on staining of lung and muscle tissue of lean and obese male mice. A Z-stack containing 21 pictures scanning vertically through the tissue was recorded from control mice as well as 192 h after trauma (*n* = 5). These 21 fields were used to calculate the mean indicating percent positive collagen area. All stainings were acquired with an Olympus IX81 and analyzed with the Olympus software cellSens Dimensions 2.3 (Build 18987).

### Gene Expression Analysis

Liquid N_2_ frozen parts of skeletal muscle of the left leg and lung tissue were crushed, dissolved in 600 μL homogenizer buffer and homogenized using a tissue homogenizer. Subsequent RNA isolation was performed with the RNeasy^®^ Mini Kit (Qiagen^®^) according to manufacturer specifications (RNeasy Mini Handbook, Protocol: Purification of total RNA from tissue, 06/2012). RNA was eluted in the final step with 30 μL nuclease-free water. Generation of cDNA via reverse transcription was performed with the AffinityScript Multiple Temperature cDNA Synthesis Kit (Agilent Technologies) according to manufacturer specifications. A total amount of 1000 ng purified RNA was used for each reaction.

In SYBR^TM^ Green-based qPCR analysis, *Tbp* (QT00198443, Qiagen) as well as *Rplp* (QT00249375, Qiagen) were used as reference genes (Gong et al., 2016). Target genes were *Mmp2* (QT00116116, Qiagen), *Mmp9* (QT00108815, Qiagen), *Timp1* (QT00996282, Qiagen), *Lox* (QT00098028, Qiagen), *Fn1* (QT00135758, Qiagen), *Col1a* (QT00162204, Qiagen), *Col3a* (QT01055516, Qiagen) and *Acta2* (QT00140119, Qiagen). In muscle, additional genes including *Myod* (QT00101983, Qiagen), *Myog* (QT00112378, Qiagen), *Myf5* (QT00199507, Qiagen) were investigated. Gene expression profiles were determined in injured lung or muscle of lean and obese control animals as well as 1, 6, 24, 72, and 192 h post-trauma via qPCR (*n* = 5). *Tbp* and *Rplp* were used as housekeeping genes. Gene expression profiles of genes are shown as fold change ± SEM. The ΔΔCT method was used for the depiction of the results. The final graph uses the depiction of the fold change and control mice were excluded from the graph, however, the control level is indicated by a dotted line. Data was analyzed using the log of the fold change and a two-way ANOVA followed by an uncorrected Fisher LSD test (*n* = 5). The log was used for statistical analysis to avoid skewness of the data.

### IHC Staining of Ki67

Paraffin-embedded, 5 μm thick cross-sections of skeletal muscle and lung tissue were dried for 24 h at 40°C with subsequent deparaffinization in Roti-Histol^®^ and hydration in decreasing alcohol series before having been used for Ki67 staining. The samples were boiled for 15 min at 450 W in a microwave using citrate demasking solution (14746, CST). After a cooling period and several washes with water as well as PBS the samples were stained overnight at 4°C with the Ki67 rabbit mAb (12202, CST) at a dilution of 1:250. The sections were washed, and the secondary staining was performed with one drop Histofin Simple Stain Max Po Anti-Rabbit (414142F, medac diagnostika). Sections were incubated for 20 min. The samples were washed and a counterstaining with hemalaun (109249, Sigma-Aldrich) was performed. Finally, sections were mounted with Entellan^®^. Two areas per animal were evaluated based on trauma region. Four animals were evaluated from each group and a picture was taken with the UC30 color camera at X10 and X40 magnification. All stainings were acquired with an Olympus IX81 and analyzed with the Olympus software cellSens Dimensions 2.3 (Build 18987). Percentage was calculated as Ki67 positive cells normalized to the total amount of cells in the field.

### Determination of *ex vivo* Muscle Force

*Ex vivo* measurement of muscle force was conducted with male mice using a modified method described by Hakim et al. (2011). Measurement was conducted with control mice as well as 24 and 192 h post trauma. Left *extensor iliotibialis anticus* was isolated and two loops were attached at the distal and the proximal tendon using surgical suture. The muscle was mounted vertically between two iron hooks. The lower hook was rigid. The upper one was connected to a force transducer and allowed to adjust the muscle tension via a micrometer screw. Force data were recorded on a computer. The construction was submerged in Ringer’s solution (118 mmol/L NaCl, 3.4 mmol/L KCl, 0.8 mmol/L MgSO_4_, 1.2 mmol/L KH_2_PO_4_, 11.1 mmol/L dextrose, 25 mmol/L NaHCO_3_, 2.5 mmol/L CaCl_2_, pH 7.4) constantly bubbled with a mixture of 95% O_2_ and 5% CO_2_. Muscle tension was set to 10 mN and equilibration was conducted for 10 min. Muscle was extracted from Ringer’s solution and muscle length was changed to generate a tension of 5 mN. Stimulation of muscle was conducted with external cardiac pacemaker at 60 Hz for 5 s with constant recording of generated muscle force. Stimulation of muscle was repeated with varying lengths generating tensions of 8, 10, 12, 15, 18, and 20 mN. The maximal twitch force (Fmax) was determined as the biggest difference between the maximum and the baseline of the recorded peaks. The length generating the maximal twitch force was referred to as optimal muscle length (*L*_*o*_) and was determined with a caliper. Muscle was removed from hooks, loops were removed and weight and dimensions of muscle were determined with a digital caliper. The data was normalized using the muscle cross sectional area (MCSA). MCSA was calculated with the following equation, adapted from Hakim et al. (2013):

$$MCSA\left[cm^{2}\right]=\frac{musclemass}{\rho_{muscle}*L_{o}}$$

$$\rho_{muscle}=1.06\frac{g}{cm^{3}}$$

### Gelatin Zymography

Fifty microgram of tissue was used for the preparation of protein extract and dissolved in 0.5 Fml ice-cold NP-40 lysis buffer (50 mM Tris-HCl, 120 mM NaCl, 1% NP-40, 10% Glycerol). The liquid N_2_ frozen parts of skeletal muscle tissue were homogenized in lysis buffer over ice for 30 min after tissue crushing with a pestle. This homogenate was centrifuged in a microcentrifuge at 16,000 × g for 20 min at 4°C. The supernatant was collected, and the protein concentration was measured with a Pierce BCA Protein Assay Kit (23225, Thermo Fisher Scientific). Ten microgram of protein was used for electrophoresis. Samples resolved in non-reducing sample buffer were loaded on a 10% acrylamide gel containing 5 mg/mL gelatin. After electrophoresis, the gel was placed in renaturing buffer (2.5% Triton X-100, 50 mM Tris-HCl pH 7.5, 5 mM CaCl_2_, 1 μM ZnCl_2_) for 30 min at RT. Subsequently, the gel was developed using developing buffer (1% Triton X-100, 50 mM Tris-HCl pH 7.5, 5 mM CaCl_2_, 1 μM ZnCl_2_) for 30 min at RT followed by a 16–18 h incubation with fresh developing buffer at 37°C. The gels were stained with Coomassie Blue staining solution, acquired with a Vilber Fusion FX and evaluated with Image J. The muscle samples of five lean and obese male mice, including control mice as well as mice after 1, 6, 24, 72, and 192 h of trauma induction, were measured in triplicates.

### LEGENDplex

BioLegend’s bead-based immunoassay LEGENDplex was performed to determine the level of several cyto- and chemokines in plasma. Plasma was obtained by centrifugation of 0.5 mL blood collected into an EDTA coated tube at 800 × g for 5 min at 4°C. A custom version of the mouse inflammation panel (740446, BioLegend) was used with a V-bottom plate. The used analytes were IL-6 (Bead B4), MCP-1 (Bead A8), TNF-α (Bead A7), and IL-23 (Bead A4). The staining and acquisition was carried out according to the manufacturer specifications (LEGENDplex^TM^, Mouse Inflammation Panel with V-bottom Plate, 05/2020). The analysis was performed with the LegendPlex analysis software v8.0.

### Statistical Analysis

Graphpad Prism 7.04 was used for statistical evaluation of the graphs. The used tests for each analysis are depicted below the graph. In general, a two-way ANOVA followed by an uncorrected Fisher’s LSD test (α = 0.05) was used if several time or diet-dependent comparisons were performed. If only specific time points were compared as it is the case for the delta change of the muscle force a one-tailed, unpaired t-test was performed (α = 0.05). Data is depicted as Mean ± SEM. Since the data of the gelatin zymography was non-parametric, an unpaired, one-tailed Mann-Whitney test (α = 0.05) was used. The following indicators were used for all statistical tests: ^∗^ indicates *p* < 0.05, ^∗∗^ indicates *p* < 0.01, ^∗∗∗^ indicates *p* < 0.001, ^****^ indicates *p* < 0.0001.

## Results

In order to investigate the influence of obesity on tissue regeneration of muscle of the left hind limb *extensor iliotibialis anticus* as well as the lung, an established combined trauma model inducing blunt injuries in 16 ± 1 week old male, lean and obese C57BL/6J mice was used (Knoferl et al., 2004) to characterize morphological changes at various time points post trauma induction (Xu P. et al., 2018) and to evaluate gene expression of genes either involved in ECM organization or satellite cell function.

**FIGURE 1:**
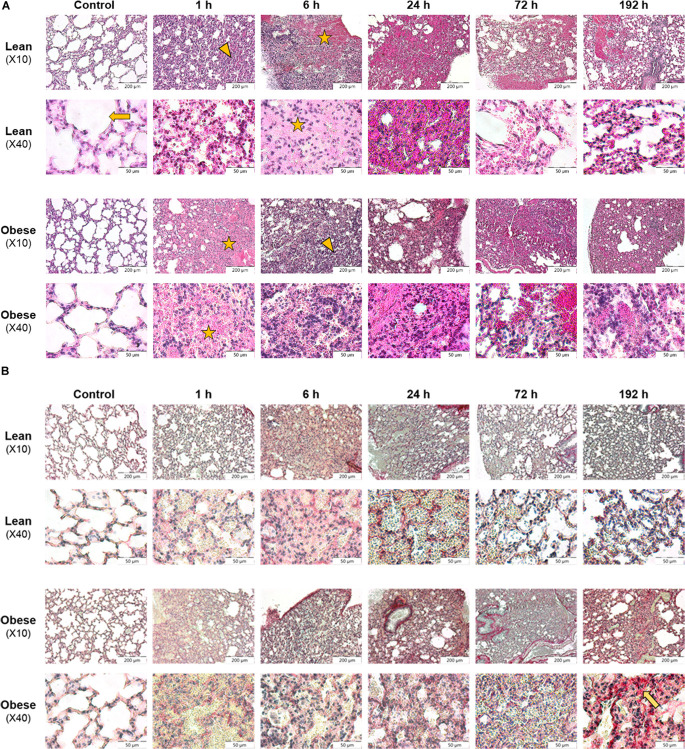
Morphological evaluation of lung shows restricted clearance of tissue debris in obese mice 192 h after trauma induction. Histology of regeneration process in lung of male lean and obese C57BL/6J mice after induction of blunt thorax trauma (*n* = 6). **(A)** Hematoxylin and eosin (HE) staining of lung tissue sections from control animals as well as 1, 6, 24, 72, and 192 h post trauma. Blunt injury led to alveolar (arrow) influx of erythrocytes (asterisk) 1 h post trauma. Further, alveolar contusion (triangle) and influx of immune cells was visible. **(B)** Sirius red (SR) staining of lung tissue sections from control animals as well as 1, 6, 24, 72, and 192 h post trauma. Interstitial collagen deposition (arrow) was visible 192 h post injury, elevated in obese animals. Pictures were taken with the UC30 color camera at X10 and X40 magnification (OLYMPUS IX81). Scale bar: 200 μm (X10), 50 μm (X40).

Damage and regeneration process in the lung based on morphological evaluation and collagen quantification is unaffected by diet. Time- and diet-dependent regeneration process of lung tissue was visualized with HE and SR staining (Figure 1) to assess the effect of injury, morphological changes and collagen deposition during the regeneration process.

Morphology of lung tissue of control animals is similar in lean and obese individuals based on visual evaluation (Figure 1A). Alveolar structure, limited by alveolar epithelial cells, was clearly visible in control animals. The induction of trauma led to a comparable reaction in lean and obese mice including pulmonary contusion, which describes the disruption of alveolar region and blood vessels. As a result, increased influx of erythrocytes (strong pink color) was visible 1, 6, and 24 h post trauma. At later time points an infiltration of immune cells (blue color) responsible for removing tissue debris was detectable 6 and 24 h post trauma respectively in lean and obese mice. In lean mice tissue debris was removed from alveoli, and rebuilding of alveolar compartment was proceeded 72 and 192 h post trauma. Nevertheless, erythrocytes were still visible 192 h post trauma, probably indicating that the regeneration process has not been completed, yet. In contrast, tissue debris in the alveoli was still visible in obese mice 192 h post trauma. Apart from that, structure of alveolar compartment had still not been regenerated. Moreover, erythrocytes were still visible in a huge area 192 h post trauma indicating that the regeneration process is far from being completed. This might indicate an impairment of the clearance of tissue debris in obese animals, which will be further assessed by conducting gene expression analysis.

**FIGURE 2:**
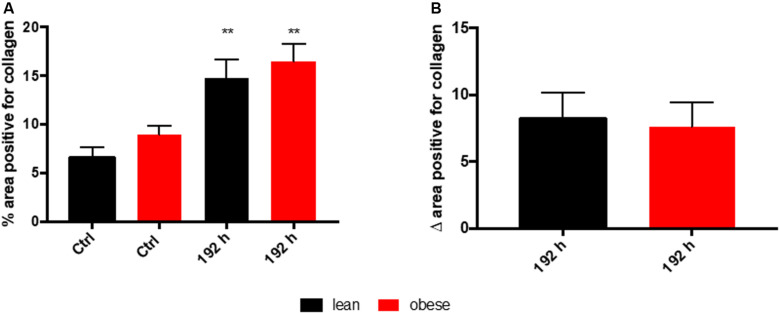
Collagen deposition in the lung as a response to trauma is independent of diet after 192 h. Determination of collagen content in the lung using picrosirius red staining based on auto-fluorescence properties of the dye. The percent of the positive areas for collagen in control mice as well as 192 h post trauma (*n* = 5) **(A)** is depicted next to a graph depicting the differences of the post trauma mice to the respective control mice **(B)**. The data is presented as mean ± SEM. Statistical significance was determined using two-way ANOVA followed by an uncorrected Fisher’s LSD test (α = 0.05). **p < 0.01.

Assessment of collagen deposition during regeneration process was achieved by an implementation of SR staining (see Figure 1B). Diet-dependent differences in collagen deposition were not detectable in control animals. Also, during the first 72 h of regeneration process, differences in collagen deposition were not visible. However, 192 h post trauma obese animals showed increased areas that are filled with interstitial ECM compared to lean animals. A densitometric analysis based on the fluorescence potency of picrosirius red was used to quantify the amount of collagen (Figure 2). Even though an increase of collagen deposition was detectable in lean and obese mice as a response to trauma (Figure 2A), no difference was detectable in the changes after 192 h between lean and obese animals (Figure 2B).

Based on the morphological assessment via HE and SR staining, the regeneration process seems not to be completed 192 h post trauma in lean and obese mice. Nevertheless, regeneration of the lung might be delayed in obese animals due to an increased deposition of ECM 192 h post trauma. Quantitative gene expression analysis was conducted to further characterize ongoing regeneration processes with a focus on ECM regulation and its structure.

### Diet-Dependent Expression Differences of Genes Encoding for ECM Structure and ECM Organizing Proteins in the Lung After Trauma

**FIGURE 3:**
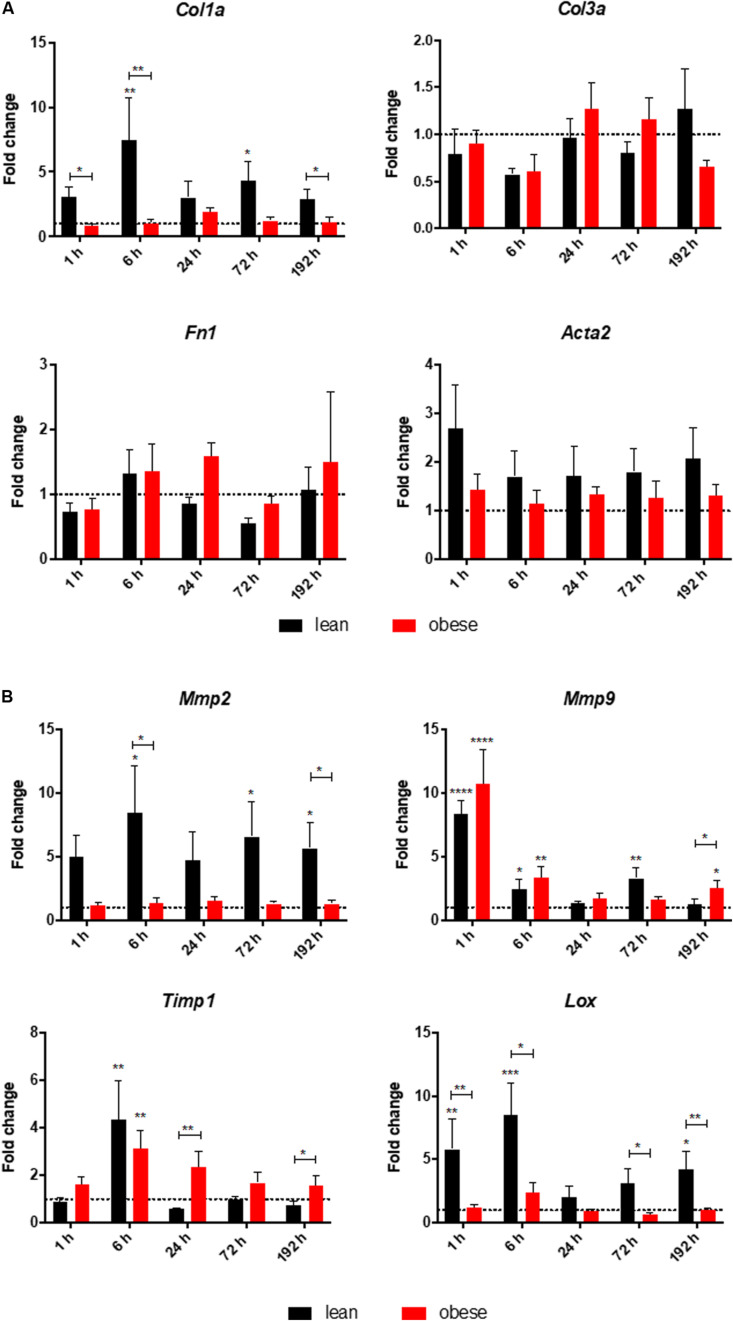
Altered response in obese mice to the trauma based on gene expression profiles of **(A)** ECM structure genes *Col1a*, *Col3a*, and *Fn1* as well as *Acta2* and **(B)** ECM organizing genes namely *Mmp2*, *Mmp9*, *Timp1*, and *Lox* in the lung. Statistical significance was determined using two-way ANOVA followed by an uncorrected Fisher’s LSD test (α = 0.05). *p < 0.05, **p < 0.01, ***p < 0.001, ****p < 0.0001. Indicators of significance directly above the bars of the diagram indicate a statistical difference to the control, while starts above the connector line show differences between the two diets at that specific time point.

A quantitative gene expression analysis was conducted to further investigate the increased interstitial ECM deposition in the lung of obese mice 192 h post trauma (Figure 3). Gene expression analysis of the three ECM structure genes *Col1a*, *Col3a*, and *Fn1* revealed that only *Col1a* seemed to be influenced by obesity during its response to the trauma (Figure 3A). *Col1a* is differentially expressed and statistically significantly decreased in obese mice 6 and 192 h after trauma. Furthermore, *Acta2* did not show any differences between lean and obese animals, although a constant increase can be detected in lean animals from 6 to 192 h, which was not visible in obese animals.

Genes being involved in organization of the ECM including Lysyl oxidase (*Lox*), *Mmp2*, *Mmp9*, and *Timp1* were determined via qPCR in lean and in obese control animals as well as 1, 6, 24, 72, and 192 h post trauma (see Figure 3B). Regarding the gene expression profile of *Lox*, a bimodal response can be observed with an early response peaking at 6 h post trauma and a late response peaking at 192 h post trauma. The late response is most likely not completed yet, but 192 h was the last observation point of this study. The gene profile was significantly decreased in obese mice compared to lean animals during the early response as well as the late response. This could hint toward a decreased stability of the newly regenerated tissue, since the crosslinking of the generated ECM is not as efficient in obese mice compared to the lean mice. In contrast to the bimodal response of *Lox, Timp1* is an early responding gene and its upregulation is peaking 6 h after the induction of the trauma in obese and lean animals. The initial response seems to be non-significantly decreased in obese animals, however this response is elongated. In contrast to lean mice, where the expression of this gene is back to baseline after 24 h, obese mice keep an upregulated level throughout the observation time. This leads to a significantly increased gene level of *Timp1* even 192 h post trauma in obese mice compared to lean mice. Timp1 is the inhibitor of various MMPs. Therefore, the gene level of *Mmp9* as well as *Mmp2* was investigated as well. *Mmp9* shows a strong early upregulation 1 h after the induction of the trauma. However, obese and lean animals manage to return to baseline quickly after the initial response. *Mmp2* in contrast did not show any response to the trauma in obese animals. This led to a decreased gene expression in obese animals compared to lean mice during all time points. This was shown to be significant 6 h as well as 192 h after trauma.

### Damage and Regeneration Process Based on Morphological Evaluation and Collagen Quantification Is Unaffected by Diet in the Muscle

In addition to lung injury, the combined trauma model also included a muscle injury induced in the muscle *extensor iliotibialis anticus* of the left hind limb.

**FIGURE 4:**
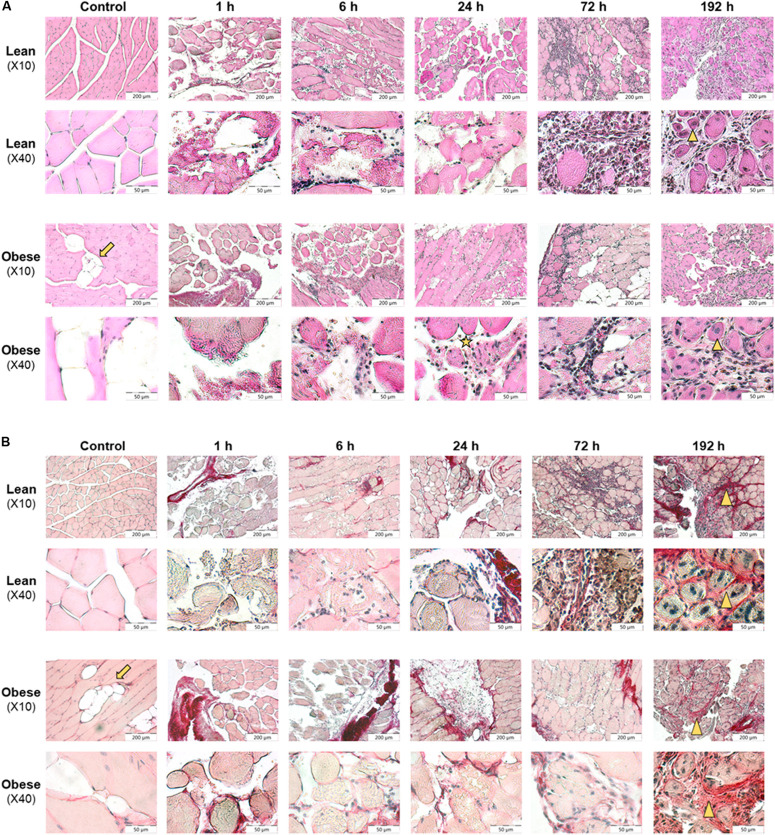
Restricted regeneration of the muscle *extensor iliotibialis anticus* and decreased density of the regenerated tissue in obese mice 192 h post trauma induction. Histology of regeneration process in muscle *extensor iliotibialis anticus* of male lean and obese C57BL/6J mice after induction of blunt injury (*n* = 6). **(A)** Hematoxylin and eosin (HE) staining of muscle tissue sections from control mice as well as 1, 6, 24, 72, and 192 h post trauma. Obese mice show fat deposition (arrow) between muscle fibers. Blunt injury led to disruption of muscle fibers and influx of erythrocytes and immune cells. Further, necrotic muscle fibers were visible (asterisk). 192 h post trauma newly formed myofibers with centered nuclei could be identified (triangle). **(B)** Sirius red (SR) staining of muscle tissue sections from control mice as well as 1, 6, 24, 72, and 192 h post trauma. Interstitial collagen deposition (triangle) was visible 192 h post injury in lean and obese mice. Pictures were taken with the UC30 color camera at X10 and X40 magnification (OLYMPUS IX81). Scale bar: 200 μm (X10), 50 μm (X40).

Comparable to the analysis of lung tissue, the timeline of the regeneration process of muscle tissue was visualized with HE and SR staining (see Figure 4). HE staining (see Figure 4A) gave a first impression about the diet-dependent muscle structure, the extent of infringement and the progression of the regeneration process. SR staining (see Figure 4B) was appraised regarding collagen deposition occurring during regeneration process.

Lean control mice showed clearly separated muscle fascicles and muscle fibers with marginal nuclei. In contrast to this, the functional, consistent muscle structure of obese mice was interrupted by fat depositions. In lean and obese mice induction of trauma led to disruption of muscle fibers and influx of erythrocytes 1–24 h post trauma. Six to twenty four hours post trauma, immune cells infiltrated the damaged tissue. Immune cells remove tissue debris and phagocytized necrotic muscle fibers. Seventy two hours post injury, a high number of cells filling in damage-related spaces was detectable. Most of these immune cells showed a distinct round shape, whereas a minority showed spindle-shaped appearance. The presence of these cells filling the interstitial space led to a compaction of the damaged tissue. 192 h post injury interstitial cells were still visible, whereas the spindle-shaped phenotype was predominant (see Figure 4A).

According to visual assessment, the density of the regenerated muscle fibers is higher in lean animals compared to obese animals. Lower density of the regenerated muscle fibers in obese animals may be caused due to the observed fat depositions and increased interstitial spaces in obese animals. Muscle fibers showed a round shape with nuclei that moved to the center indicating that fibers undergo a stage of regeneration. Although muscle fibers were detected to undergo a stage of regeneration 192 h post trauma, total recovery comparable to structure of control animals was not achieved in both groups (see Figure 4A).

**FIGURE 5:**
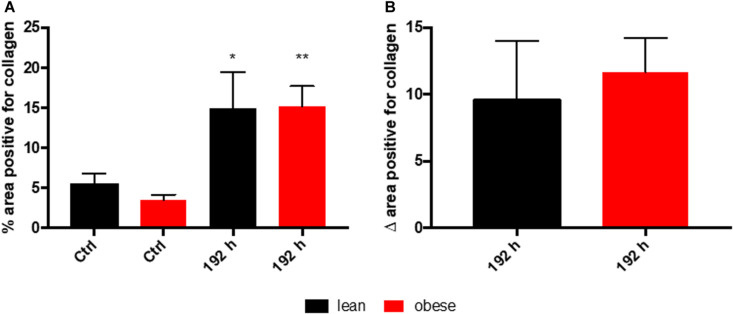
Collagen deposition in the muscle as a response to trauma is independent of diet 192 h post trauma induction. Determination of collagen content in the muscle using picrosirius red staining based on auto-fluorescence properties of the dye. The percent of the positive areas for collagen in control mice as well as 192 h post trauma (*n* = 5) **(A)** is depicted next to a graph depicting the differences of the post trauma mice to the respective control mice **(B)**. The data is presented as mean ± SEM. Statistical significance was determined using two-way ANOVA followed by an uncorrected Fisher’s LSD test (α = 0.05). *p < 0.05, **p < 0.01.

According to the evaluation of SR staining (see Figure 4B) collagen deposition was comparable in lean and obese mice during the regeneration process. Small amounts of collagen were detectable in occurring spaces caused by degraded muscle fibers starting at 72 h post trauma. 192 h post trauma, the regenerating area showed massive interstitial collagen deposition in both, lean and obese mice. However, it becomes apparent that the muscle of lean mice is more compact based on the visual evaluation of the density of the regenerated muscle. The data presented in Figure 5 shows the quantification of collagen in the muscle of control animals as well as 192 h after trauma. An increase of collagen deposition due to the trauma can be observed in lean as well as obese animals. However, no difference in the amount of deposited collagen is detectable between the two diets. This indicates that the compaction of the muscle in lean mice is not based on the influence of collagen.

Visual evaluation of muscle regeneration process in lean and obese mice showed massive fat deposition in muscle of obese mice, which might also impede the compaction of the muscle in later stage of regeneration.

### Decreased Level of ECM-Structure Genes in the Muscle of Obese Mice in Later Stages of the Regeneration Process

**FIGURE 6:**
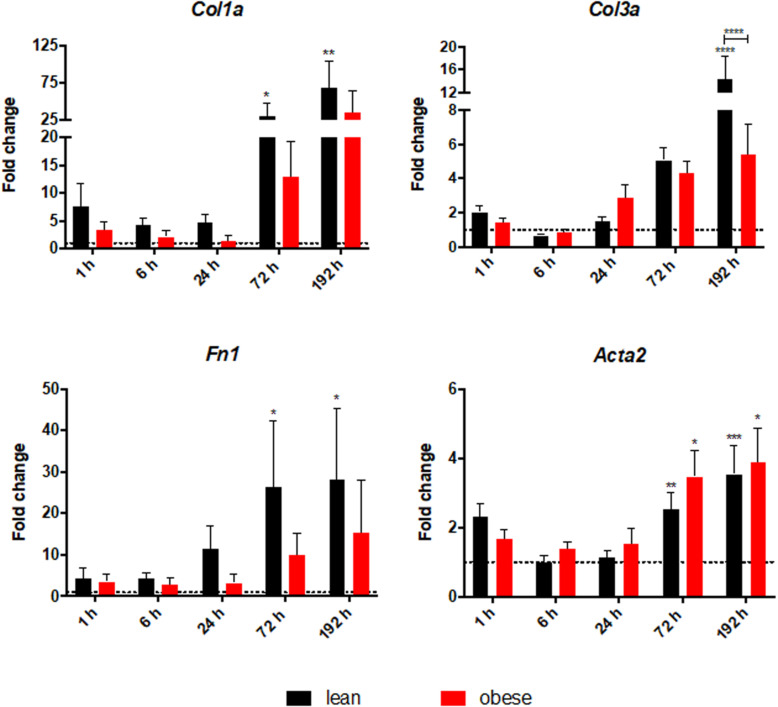
Altered level of ECM structure genes in obese mice. Gene expression profile of ECM structure genes *Col1a*, *Col3a*, and *Fn1* as well as *Acta2* were determined in injured muscle of lean and obese trauma and control animals. Statistical significance was determined using two-way ANOVA followed by an uncorrected Fisher’s LSD test (α = 0.05). *p < 0.05, **p < 0.01, ***p < 0.001, ****p < 0.0001. Indicators of significance directly above the bars of the diagram indicate a statistical difference to the control, while starts above the connector line show differences between the two diets at that specific time point.

Quantitative gene expression analysis was conducted to further characterize the ongoing regeneration processes with a focus on ECM structure (Figure 6). *Acta2* was analyzed as a marker for the formation of myofibroblasts (Nagamoto et al., 2000). The gene analysis showed a diet-independent upregulation 72 and 192 h post trauma. However, no difference between the two diets can be detected, indicating a comparable level of myofibroblast formation as a response to the trauma in lean and obese mice.

*Col1a*, *Col3a*, and *Fn1* were analyzed to access the level of genes that encode proteins building up the ECM. All three genes show a statistically significant increase in lean mice 72 h as well as 192 h post trauma. A trend of increasing levels of those genes in the last two time points can also be seen in obese animals, but without statistical significance. The lower magnitude of response in obese animals to the trauma results in a statistical increased level of *Col3a* after 192 h post trauma in lean animals compared to obese animals. These results contrast with the observation of comparable collagen deposition between the two diets. This hints toward an additional regulatory step of ECM organization that is differentially regulated in obese and lean mice to explain the higher gene level of collagens and fibronectin. In combination with the gene analysis of *Acta2* this indicates that although formation of the myofibroblasts is comparable between lean and obese mice the activity of myofibroblasts must be different. Myofibroblasts are differentiated cells, that are responsible for the production of collagens while having the ability to contract (Lowe and Anderson, 2015). Therefore, same formation of these cells accompanied by a decreased production of collagen hints toward a decreased activation of these cells. However, this also hints toward another level of ECM regulation since the endpoint determination of collagen deposition showed no difference between the two diets. Therefore, genes that give insight into the ECM organization were characterized.

### Decreased Expression of ECM Organizing Factors in the Muscle of Obese Mice

**FIGURE 7:**
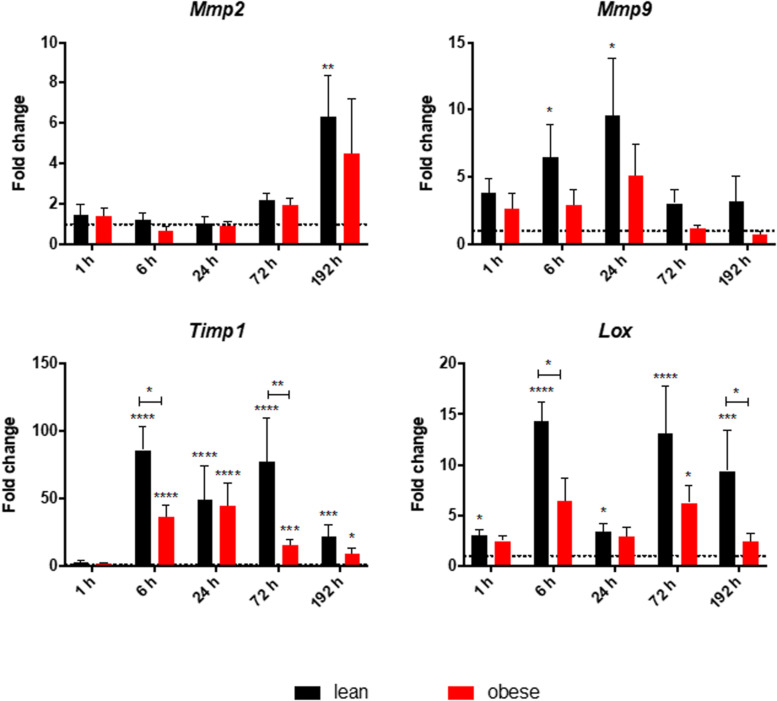
Decreased early and late response of gene level of ECM organizing genes in obese mice. Gene expression profile of ECM organizing genes *Mmp2*, *Mmp9*, *Timp1*, and *Lox* were determined in injured muscle of lean and obese control and trauma animals. Statistical significance was determined using two-way ANOVA followed by an uncorrected Fisher’s LSD test (α = 0.05). *p < 0.05, **p < 0.01, ***p < 0.001, ****p < 0.0001. Indicators of significance directly above the bars of the diagram indicate a statistical difference to the control, while starts above the connector line show differences between the two diets at that specific time point.

Gene expression of *Lox*, *Timp1*, *Mmp2*, and *Mmp9* was determined to investigate the ongoing mechanisms regarding ECM organization after combined traumatic injury (Figure 7). Assessment of gene expression profile of *Lox* indicated a significant upregulation in lean mice during all time points post injury. The trend followed an early response peaking 6 h after trauma and a late response peaking 72 h after trauma. This bimodal response curve was also detectable in obese mice, but in a much lower magnitude, which led to a statistically significant decrease of the early response (6 h) and late response (192 h) in obese mice. The decreased level of *Lox*, encoding for an enzyme responsible for crosslinking collagen and elastin (Kothapalli and Ramamurthi, 2009), might hint toward a decreased build-up of ECM as a response to the trauma. This would result in an instable matrix and decreased stability resulting in an impaired regeneration and decreased muscle force. Besides crosslinking proteins like LOX, degrading enzymes like MMPs and their inhibitors TIMPs are also important for ECM organization. Gene expression of *Timp1* significantly increased in lean mice starting at 6 h post trauma throughout the observed regeneration process. This gene also follows a bimodal expression profile peaking at 6 and 72 h. This bimodal expression profile cannot be observed in obese animals leading to statistically significant downregulated levels in these mice at 6 and 72 h post trauma. Nevertheless, obese mice also show a statistically increased level of *Timp1* throughout the regeneration process after 6 h. However, they are showing a unimodal profile peaking at 24 h post trauma. Increased levels of *Timp1*, encoding for an enzyme inhibiting the activity of various MMPs, hint toward a decreased MMP activity, resulting in an increased build-up of the ECM. Regarding the gene expression of *Mmp2*, an increase was detectable as response to the trauma in lean and obese mice after 192 h. However, this difference was only significant in lean mice. Expression of *Mmp2* starts to continuously increase in mice between 24 and 192 h post trauma, resulting in a late response to the trauma. Assessment of gene expression of *Mmp9* showed significant upregulation in lean mice 6 and 24 h post trauma. In obese mice, the magnitude of the expression profile of *Mmp9* follows lean mice, but always in a weaker and non-significant manner. This gives *Mmp9* the role of an early responsive gene to the trauma. Gelatin zymography was performed to investigate the activity of MMP9 and MMP2 in obese and lean animals and translate the gene research to the protein level showing that no significant differences in the activity of MMP9 and MMP2 are detectable in lean and obese mice at the indicated time points after combined trauma induction (Supplementary Figure S1). However, the activity of MMP9 and MMP2 followed the gene profile, with MMP9 being upregulated to control in the earlier time points after trauma, while MMP2 being responsive to the trauma at later time points. 192 h after trauma this leads to a decreased activity of MMP2 in obese mice compared to lean mice, although this difference is not statistically significant.

Analysis of gene expression profiles of various genes involved in ECM-organization showed a bimodal response of *Lox* and *Timp1* peaking at 6 and 72 h, while being significantly decreased in obese mice compared to lean mice. Additionally, a decreased expression level of *Mmp9* and *Mmp2* could be detected in obese mice, which translates to the protein level with a decreased level of MMP2 activity after 192 h. The changed expression profile in obese mice hint toward a weakened crosslinking of the ECM, which could result in a decreased stability and recovered muscle force in those animals.

### Decreased Activation of Satellite Cells During Regeneration in the Muscle in Obese Animals

**FIGURE 8:**
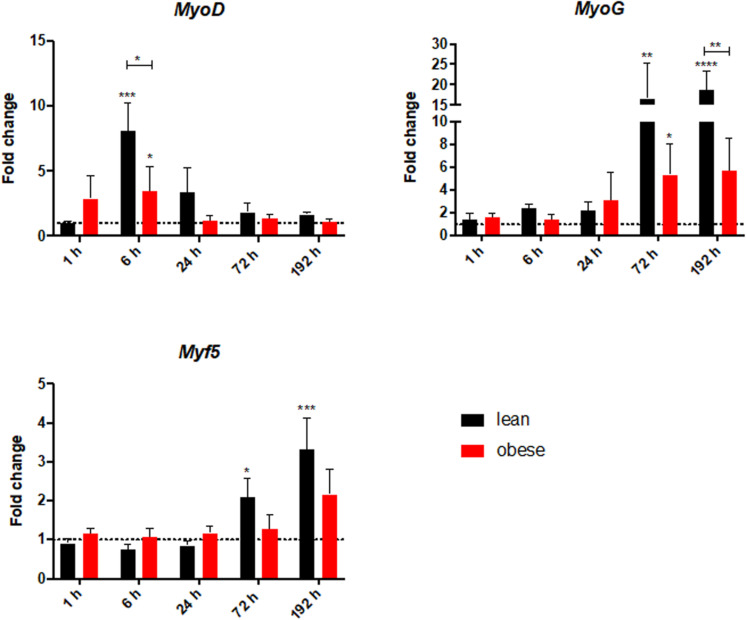
Decreased level of satellite cell activation in obese mice based on gene expression profile of satellite cell genes *MyoD*, *MyoG*, and *Myf5*. These levels were determined in injured muscle of lean and obese control and trauma animals. Statistical significance was determined using two-way ANOVA followed by an uncorrected Fisher’s LSD test (α = 0.05). *p < 0.05, **p < 0.01, ***p < 0.001, ****p < 0.0001. Indicators of significance directly above the bars of the diagram indicate a statistical difference to the control, while starts above the connector line show differences between the two diets at that specific time point.

**FIGURE 9:**
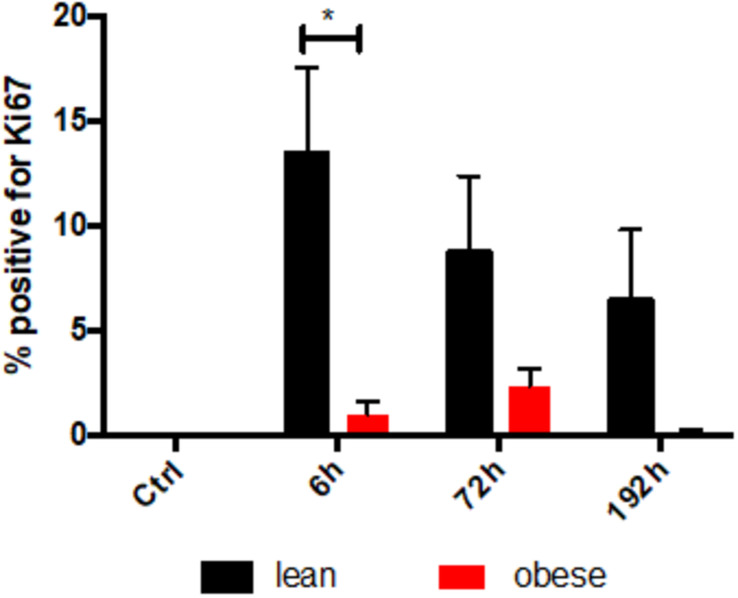
Decreased proliferationin the tissue after trauma induction based on IHC staining of KI67 in obese animals. Ki67 stainings were evaluated for the percentage of proliferating cells in the trauma muscle in lean and obese control mice as well as 6, 72, and 192 h post trauma. The data is presented as mean ± SEM (*n* = 4). Statistical significance was determined using two-way ANOVA followed by an uncorrected Fisher’s LSD test (α = 0.05). *p < 0.05. Indicators of significance directly above the bars of the diagram indicate a statistical difference to the control, while starts above the connector line show differences between the two diets at that specific time point.

Gene expression analysis was focused on satellite cells to further investigate differences in regeneration after blunt combined trauma in the muscle of obese and lean mice. Expression levels of satellite cell markers *Myf5*, *Myod*, and *Myog* were determined in lean and obese control mice and 1, 6, 24, 72, and 192 h post trauma respectively (see Figure 8). Gene expression profiling of *Myf5* showed a late response to the trauma with increased expression values after 72 and 192 h in lean mice. The fold change in obese mice follows this trend but stays non-significantly below the lean mice. Although there was no significant difference between the diets detectable in expression profile of *Myf5*, changes in the fold changes of the satellite cell markers *Myod* and *Myog* indicated diet-dependent differences in response to injury. Gene expression of *Myod* was significantly upregulated in lean and obese mice 6 h post trauma. However, the response in obese mice was statistically significantly decreased compared to lean mice. This trend of reduced activation of satellite cells in the obese muscle is continued with the expression profile of *Myog*, a marker for committed satellite cells. Lean and obese mice upregulate this gene significantly after 72 h as a response to trauma. Nevertheless, this response is decreased in obese mice leading to a statistically significant decrease after 192 h in comparison to the lean mice. This indicates a decreased activation as well as commitment based on the gene profile and hints toward a limited trauma response and could further explain the observed differences in regeneration of the muscle in obese and lean mice after blunt muscle trauma. This decreased activation and limited response is also noticeable among the percentage of dividing cells in the muscle. A Ki67 staining was performed to evaluate the percentage of dividing cells in the trauma region. The percentage of dividing cells is increased in both lean and obese mice as a response to trauma, however, this response is statistically significantly lower in obese mice (Figure 9). The according pictures of the Ki67 staining can be found in Supplementary Figure S2. The decreased percentage of dividing cells in obese mice coincides with the finding of deregulated activation and commitment based on genes of satellite cells and strengthens the theory of decreased stability and recovery of the muscle strength after injury in those mice.

### Decreased Level of Regained Muscle Force in Obese Animals After Blunt Muscle Trauma

**FIGURE 10:**
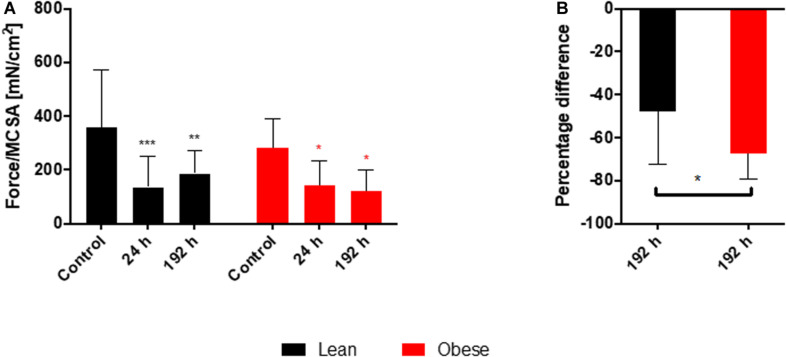
Decreased recovery of the muscle force in obese animals 192 h post trauma. Evaluation of muscle force during regeneration process of injured muscle extensor iliotibialis anticus of lean and obese control mice as well as 24 and 192 h post trauma (*n* = 6). **(A)** Time-dependent development of muscle force normalized to muscle cross sectional area (Force/MCSA [mN/cm^2^] ± SEM). Statistical significance was determined using two-way ANOVA followed by an uncorrected Fisher’s LSD test (α = 0.05). **(B)** Comparison of Force/MCSA [mN/cm^2^] between lean and obese mice 192 h post trauma normalized to the respective control. Statistical significance was determined with an unpaired, one-tailed *t*-test (α = 0.05). *p < 0.05, **p < 0.01, ***p < 0.001.

The decreased activation and differentiation of the satellite cells in obese mice after trauma as well as the analysis of several genes involved in the organization and the build-up of ECM hints toward a decreased stability of the muscle after injury, which would result in a decreased recovery of muscle strength. This theory of restricted regenerative capacity was approached with the determination of the muscle force in lean and obese control mice 24 h as well as 192 h post trauma (see Figure 10). Time-dependent development of muscle force normalized to MCSA (see Figure 10A) showed a significant, diet-independent decrease after 24 h as a result to blunt-injury. However, the recovery of this muscle force after trauma is different between lean and obese mice. 192 h post trauma muscle force started to increase in lean mice, whereas in obese mice an increase was not detectable. Muscle force determined 192 h post trauma was separately compared between lean and obese mice normalized to the muscle force of the respective control mice (see Figure 10B) to specify this difference showing that the recovery of muscle strength is statistically significantly decreased in obese mice compared to lean mice.

### Increased Level of Inflammatory Cyto- and Chemokines in Obese Animal After 192 h in the Plasma

**FIGURE 11:**
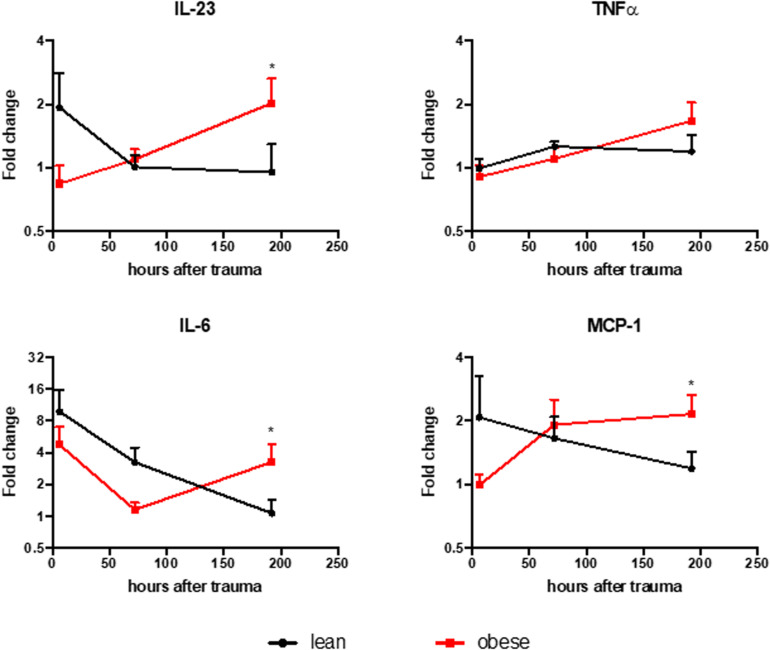
Lean animals show resolved inflammation 192 h post trauma induction, while obese mice show elevated levels of IL-23, MCP-1, and IL-6. Legendplex assay was performed to evaluate the level of IL-23, MCP-1, IL-6, and TNFa in the plasma of lean and obese mice. The level of chemo- and cytokines were determined in control mice as well as 6, 72, and 192 h post trauma. The data is normalized to the respective control group and presented as mean ± SEM. The comparison was made between the two diets at each time point using two-way ANOVA followed by an uncorrected Fisher’s LSD test (α = 0.05). *p < 0.05.

The level of several pro-inflammatory chemo- and cytokines were measured in the plasma of lean and obese animals to support the findings of an elongated and changed response to trauma in obese mice as it was suggested by the gene expression profile of several genes investigated in this study. The results were normalized to the respective control group (Figure 11). Obese mice did not resolve the inflammation that occurs as a response to the trauma 192 h after trauma. Several factors are still upregulated in obese animals compared to lean mice at that time point, indicating that the inflammation is still ongoing. This can influence the regeneration process of both lung and muscle since the inflammatory milieu can affect the switch of M1 to M2 macrophages. This switch is especially important since M2 macrophages are main drivers in the degradation of collagen to prevent a possible fibrosis (Madsen et al., 2013). Additionally, M1 macrophages directly hinder the activation of satellite cells (Perandini et al., 2018). Therefore, a prolonged inflammatory milieu could explain the reduced commitment level of satellite cells in early stages based on *Myod* and the decreased activation at later stages based on *Myog* in obese mice.

## Discussion

Obesity is associated with an abnormal accumulation of fat in adipose tissue, negatively influencing its function (Longo et al., 2019). This dysfunction is characterized by an altered secretion of adipocytokines (Leal Vde and Mafra, 2013) and inflammatory cytokines (Monteiro and Azevedo, 2010) as well as a restricted lipid metabolism (Sam and Mazzone, 2014), influencing whole body metabolism. In this study the effects of DIO on regeneration of lung and muscle after induction of blunt combined traumatic injury were investigated.

Visual assessment of lung regeneration via HE and SR staining showed delayed regeneration process with higher build-up of interstitial ECM in lung of obese mice 192 h post trauma (Figure 1). However, the level of collagen, which is one of the most abundant components of lung ECM (Balestrini and Niklason, 2015), is not changed in between lean and obese mice (Figure 2). In contrast, the expression levels of proteins involved in the structure as well as the organization of ECM were indicating obesity-associated changes (Figures 3, 4). Both, genes encoding for the structure as well as the organization of the ECM, were deregulated hinting toward a decreased stability of the regenerating tissue. Noticeable in this context was the bimodal response of *Lox*, a gene encoding for an ECM crosslinking enzyme. This gene was statistically significantly decreased in obese mice during the early and late response to the trauma. *Timp1* showed an early upregulation 6 h after trauma induction in both diets. However, the fold change returned to baseline already after 24 h post trauma in lean mice, while obese mice had a significantly increased level throughout the observation time, indicating an elongated response in these mice. The effect of an elongated response can also be seen when analyzing inflammatory factors in the blood. 192 h after the induction of the trauma, elevated levels of pro-inflammatory cyto- and chemokines like IL23, IL6, and MCP-1 are still observable in obese mice indicating ongoing inflammation. Since the inflammation needs to be resolved to start the regeneration process this might be responsible for the increased *Timp1* expression. Additionally, increased levels of TIMP1 during inflammation have been reported before (Ulrich et al., 2010). Regarding the evaluation of MMP encoding genes *Mmp2* and *Mmp9* were analyzed. *Mmp9* is an early responding gene and is upregulated in obese and lean mice 1 h after trauma. However, a small, but significant increase 192 h after trauma can be detected in obese mice. This increase is significant in comparison to obese control mice as well as lean mice after trauma. This is intriguing in context with MMP9 being a regulator of the cellular response toward inflammation as it has been shown in a mouse model investigating bone repair (Wang et al., 2013). However, the role of an early responding gene has been shown before in a broad range of lung injuries in different animal models (Yaguchi et al., 1998; Gushima et al., 2001; Tan et al., 2006; Villalta et al., 2014). In a bleomycin-induced fibrotic lung model in rabbits, MMP9 was upregulated in the first response to injury followed by a more chronic upregulation of MMP2 in later stages (Yaguchi et al., 1998). Apart from that, upregulation of MMP9 was described to be closely connected to the neutrophil influx after injury (Yaguchi et al., 1998; Keck et al., 2002; Bradley et al., 2012), whereby MMP9 seems to be involved in disruption of alveolar epithelial membrane enabling neutrophil migration (Yaguchi et al., 1998; Hsu et al., 2015).

Morphological evaluation of muscle regeneration process indicated differences in muscle density, especially at later time points which might be a result of intramuscular fat deposition. Determined gene expression levels of ECM structure as well as organizing proteins revealed significant changes between lean and obese mice. The level of *Acta2* was comparable throughout the observed regeneration process between the two diets and is upregulated during later time points. *Acta2* is a gene encoding for alpha smooth muscle actin (αSMA) and is used for the determination of myofibroblast formation (Shinde et al., 2017). The formation of these cells can be assumed to be unaltered by the HFD. However, the activity of myofibroblasts seems to be decreased in obese mice, since the level of genes encoding for ECM structure proteins are decreased during the late response to the trauma. The reduced build-up of collagen and ECM is the first contribution to a reduced recovery of muscle strength in obese mice after the regeneration period of 192 h, as it was shown during this study.

This is accompanied by a changed gene profile of ECM organizing proteins. The level of *Mmp9* as an early responder and *Mmp9* as a late responder gene was comparable between the two diets, although obese animals always stayed below the level of lean mice. It was also shown in this study that these gene expression profiles were translatable to the protein activity level, since a gelatin zymography was performed for MMP9 and MMP2. An increased level of activity was observed during the earlier time points for MMP9, while MMP2 activity increased after 192 h as a response to the trauma. However, *Timp1* encoding for an inhibitor of MMPs and *Lox* encoding for an ECM cross-linking enzyme, were deregulated during the response to the trauma in obesity. Both genes showed a bimodal response to the trauma in lean mice, peaking after 6 h and after 72 h. In contrast to this, both genes were downregulated in obese mice during this early response as well as during the late response. The activity of both genes enhances the crosslinking of the ECM, therefore a decrease of these genes would indicate a reduced stability of the generated ECM and results in the second contribution to a decreased muscle force recovery in obese mice.

The third and final contribution to the decreased recovery of the muscle force in obese mice after 192 h post trauma can be found during the analysis of satellite cell genes, which are responsible for regeneration of the skeletal muscle after injury (Flamini et al., 2018). *Myod*, *Myog* as well as *Myf5* can be used to differentiate between the different states of satellite cell activation. *Myf5* is described as an early marker expressed in activated and committed satellite cells (Yin et al., 2013). During the state of commitment *Myod* is expressed (Meadows et al., 2011), while *Myog* is expressed at later time points during the migration and alignment of myoblasts to myocytes (Yamamoto et al., 2018). Although *Myf5* should be the first of the stem cell genes to respond to the injury, the gene was increased during later time points, an early response seemed to be absent. *Myf5* was shown to be an important factor in the maintenance and replenishment of the muscle stem cell population which might explain the upregulation in later time points (Gunther et al., 2013). The earliest response to the trauma can be observed 6 h after trauma induction in the expression of *Myod* which shows a significant increase in both diets, but the obese mice show a significantly decreased response. Additionally, *Myog* is increased 72 and 192 h post injury, with a significantly decreased response in obese mice These differences again hint toward a restricted response to injury in the muscle of obese mice and a constrained response amongst satellite cells, which can lead to the observed functional changes of the regenerated muscle between lean and obese animals.

The functional regeneration process was investigated by measuring the muscle force. Determination of muscle force showed a diet-independent drop in muscle force normalized to MCSA 24 h after trauma in both groups. However, lean mice started to recover from the injury 192 h after injury as the muscle force starts to increase. This effect is not observable in obese mice, where the muscle force is still decreasing 192 h post injury. This leads to a statistically significant difference between lean and obese mice 192 h after trauma. Since it has been shown that this model produces the same impact during trauma between obese and lean mice (Werner et al., 2018), lean mice showed higher regenerative capacity of muscle force compared to obese mice. According to this, the detected differences in gene expression during the regeneration process might influence the later outcome expressed by lower functionality.

## Conclusion

In conclusion, this study showed the influence of obesity in the organization of the ECM in a combined trauma model in the lung and the muscle. In the lung, this influence is detectable in a visual assessment as well as during gene expression analysis of genes encoding for ECM structure and organizing proteins. The gene analysis revealed a prolonged response to the traumatic injury in obese mice, which is also supported by increased levels of pro-inflammatory cyto- and chemokines in obese mice after 192 h post trauma, indicating that the inflammation as a response to the trauma is not resolved yet.

Additionally, the results presented in this study showed variances between lean and obese mice in the regeneration process of the muscle. Visual assessment showed an increased deposition of fat in the muscle of obese mice in the control state as well as during regeneration after trauma. It was shown that decreased gene levels of ECM structure proteins are detectable in obese mice as a response to the trauma. This is accompanied by decreased levels of genes responsible for encoding proteins that help to build-up ECM. Additionally, gene analysis revealed a decreased activation and differentiation of satellite cells in obese mice. Both observations, the decreased stability due to deregulated ECM build-up as well as the decreased regeneration by satellite cells, contribute to a decreased restoration of the muscle force in obese mice after trauma.

These observation offers several targets for investigating the influence of obesity on the regeneration of tissue after blunt traumatic injury. On the one side, focus can be put on the prolonged response due to inflammatory processes. On the other side, the decreased activation of satellite cells might be a pathway that can be targeted to ensure a better treatment of obese trauma patients. Latter is especially translatable to the human, since a decreased activation of satellite cells during obesity has been described before (D’Souza et al., 2015; Fu et al., 2016). Although the mechanism behind the decreased activation remains unclear, this observation has now been made in several obese mouse studies, including muscle dystrophy, cardiotoxin models and during this study after blunt injury in a mouse model using a combined trauma. It needs to be investigated, how this research is translatable to the human and can be utilized for patients to benefit from it. However, an attenuated response by satellite cells during type 2 diabetes mellitus has been shown in humans before (Deshmukh et al., 2015; Yoon et al., 2015). Differences in the activation potential of satellite cells during obesity might therefore not only be observable in the mouse model, but also in the human. Additionally, increased inflammation in obese patients is a well-known fact, since obesity in general is seen as a chronic inflammation (Trayhurn and Wood, 2004; Galic et al., 2010). Therefore, the reported findings including prolonged response to the trauma, due to the ongoing inflammation, might be translatable to the human patient as well.

Even though satellite impairment, prolonged response to the trauma and increased inflammation in obese mice were presented as separate findings in this study, once translated to the human this becomes one topic that needs to be targeted, since a chronic inflammation certainly impacts the regeneration of any part of the body and has a huge systemic impact on regeneration of lung or the muscle.

## Data Availability Statement

The raw data supporting the conclusions of this article will be made available by the authors, without undue reservation, to any qualified researcher.

## Ethics Statement

All mouse experiments were approved by the local and state authorities (Ulm University/license number: 1183) and carried out in accordance with local regulations and ARRIVE guidelines.

## Author Contributions

MW and UK performed the study design, funding, and supervision. PX and FG performed the animal experiments. AG performed the tissue morphological analysis and gene expression analysis. FG performed the collagen quantification as well as the Legendplex assay. PX performed the zymography. MH and AG performed the muscle force experiment. AG and FG wrote the manuscript. All authors read the final manuscript and approved it.

## Conflict of Interest

The authors declare that the research was conducted in the absence of any commercial or financial relationships that could be construed as a potential conflict of interest.

## Supplementary Material

FIGURE S1 Level of MMP2 and MMP9 activity follows the trend of gene profiling in lean and obese mice without showing significant differences between the diets. Gelatin zymography was performed to determine MMP2 and MMP9 activity in the muscle of lean and obese mice, in control mice as well as 1, 6, 24, 72, and 192 h post trauma (*n* = 5). The data is normalized to the individual control by subtracting the baseline value of the control group from the determined band intensity of the individual time points.

FIGURE S2 Decreased number of Ki67 positive cells in regenerated muscle after trauma induction in obese mice based on IHC staining. Ki67 staining in muscle *extensor iliotibialis anticus* of male lean and obese C57BL/6J mice after induction of blunt injury (*n* = 4). Ki67 staining was performed in obese and lean control mice as well as 6, 72, and 192 h post trauma. Obese mice show fat deposition (arrow) in between muscle fibers. Exemplary Ki67 positive cells are indicated with a triangle. Pictures were taken with the UC30 color camera at X10 and X40 magnification (OLYMPUS IX81). Scale bar: 200 μm (X10), 50 μm (X40).

## Abbreviations

DIO: Diet-induced obesity
ECM: Extracellular matrix
HE: Hematoxylin and eosin
IL-1β: Interleukin 1β
IL-6: Interleukin 6
Lox: Lysyl oxidase
MMP: Matrix metalloproteinases
Fmax: Maximal twitch force
MCP-1: Monocyte chemoattractant protein-1
MCSA: Muscle cross sectional area
MYOD: Myoblast determination protein 1
MYF5: Myogenic factor 5
MYOG: Myogenin
*L*_*o*_: Optimal muscle length
PAX7: Paired-box protein Pax7
SR: Sirius red
TIMPs: Tissue inhibitors of matrix metalloproteinases
TNF-α: Tumor necrosis factor alpha.
