# Supplementary figures and images for: Influence of Obesity on the Organization of the Extracellular Matrix and Satellite Cell Functions After Combined Muscle and Thorax Trauma in C57BL/6J Mice
Source: Front Physiol. 2020 Jul 28;11:849. doi: 10.3389/fphys.2020.00849 (PMC7399228; doi:10.3389/fphys.2020.00849)

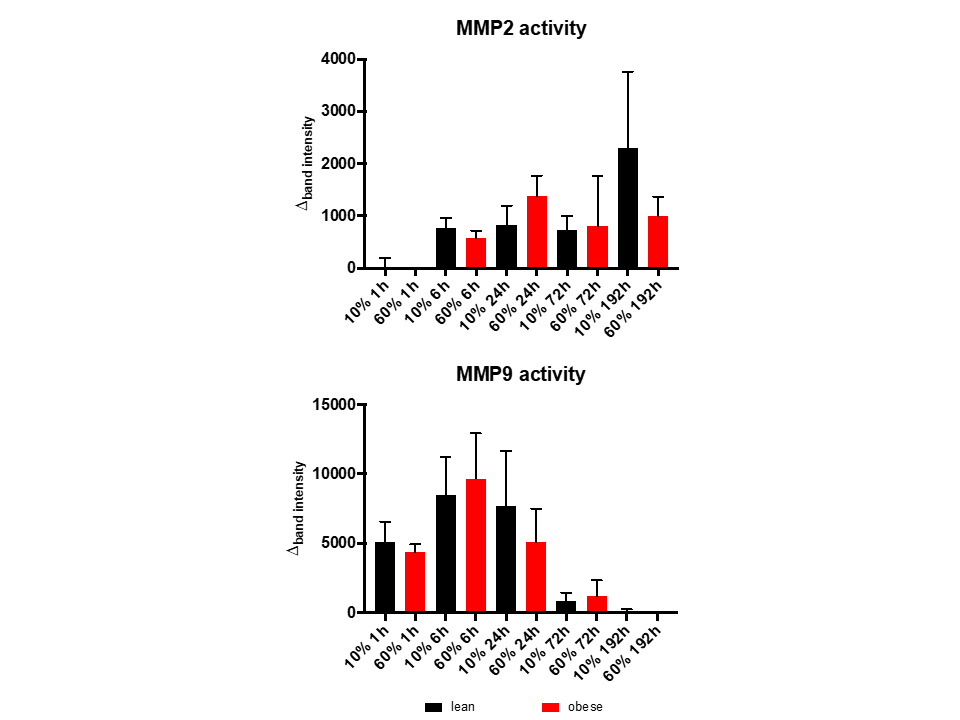

Supplement: FIGURE S1 — Level of MMP2 and MMP9 activity follows the trend of gene profiling in lean and obese mice without showing significant differences between the diets. Gelatin zymography was performed to determine MMP2 and MMP9 activity in the muscle of lean and obese mice, in control mice as well as 1, 6, 24, 72, and 192 h post trauma (n = 5). The data is normalized to the individual control by subtracting the baseline value of the control group from the determined band intensity of the individual time points. [file Image_1.TIF]

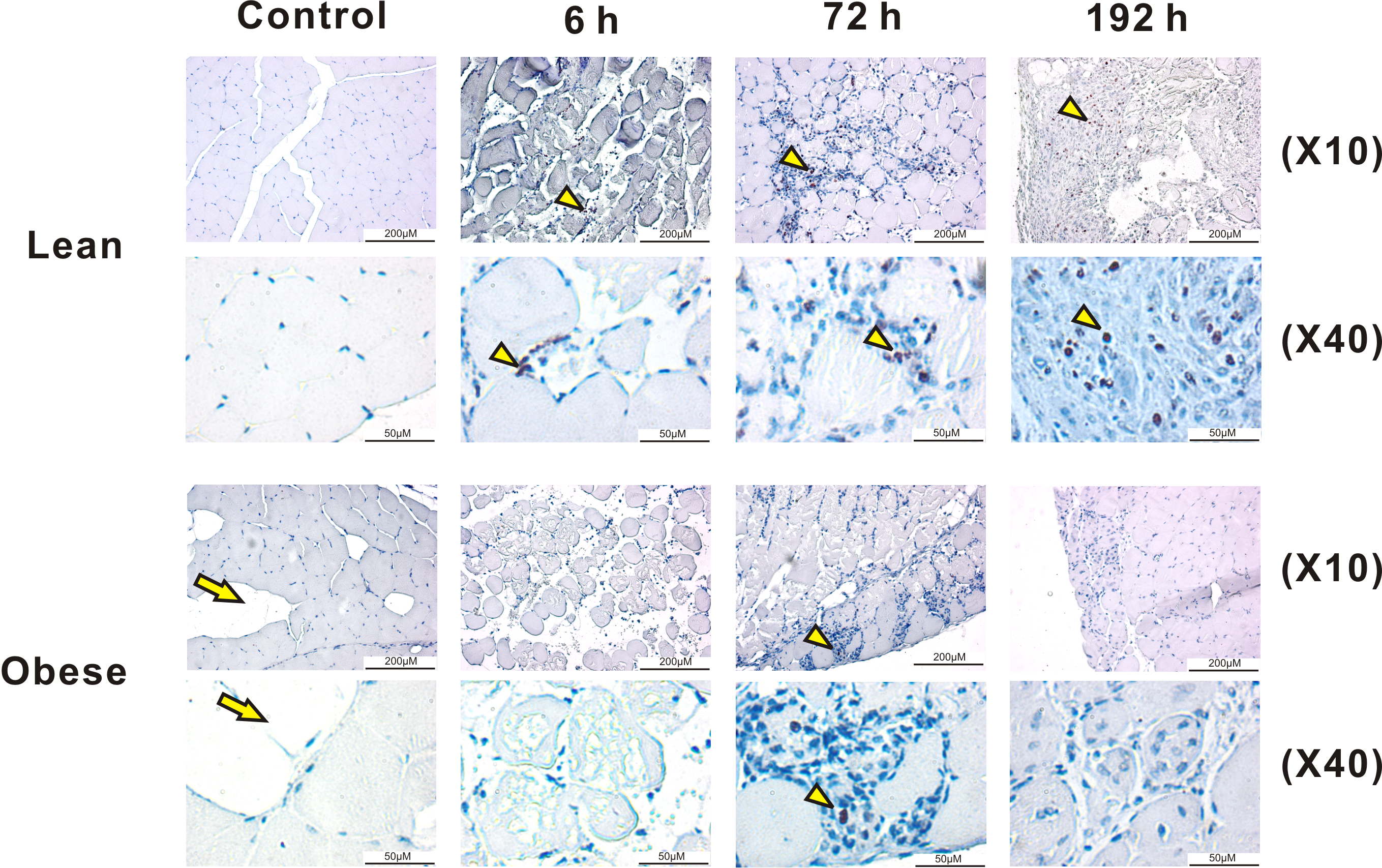

Supplement: FIGURE S2 — Decreased number of Ki67 positive cells in regenerated muscle after trauma induction in obese mice based on IHC staining. Ki67 staining in muscle extensor iliotibialis anticus of male lean and obese C57BL/6J mice after induction of blunt injury (n = 4). Ki67 staining was performed in obese and lean control mice as well as 6, 72, and 192 h post trauma. Obese mice show fat deposition (arrow) in between muscle fibers. Exemplary Ki67 positive cells are indicated with a triangle. Pictures were taken with the UC30 color camera at X10 and X40 magnification (OLYMPUS IX81). Scale bar: 200 μm (X10), 50 μm (X40). [file Image_2.TIF]
